# Understanding emotion dysregulation from infancy to toddlerhood with a multilevel perspective: The buffering effect of maternal sensitivity

**DOI:** 10.1017/S0954579424000774

**Published:** 2024-04-29

**Authors:** Mindy A. Brown, Mengyu (Miranda) Gao, Jennifer Isenhour, Nila Shakiba, Sheila E. Crowell, K. Lee Raby, Elisabeth Conradt

**Affiliations:** 1 Brigham Young University, Provo, UT, USA,; 2 Department of Psychology, Beijing Normal University School of Psychology, Beijing, China,; 3 Department of Psychology, University of Utah, Salt Lake City, UT, USA,; 4 Department of Psychology, Boston University, Boston, MA, USA,; 5 Psychology, University of Oregon, Eugene, OR, USA; 6 Psychology, Duke University, Durham, NC, USA

**Keywords:** behavioral affect, infant emotion dysregulation, maternal sensitivity, respiratory sinus arrhythmia, still-face paradigm

## Abstract

Challenges with childhood emotion regulation may have origins in infancy and forecast later social and cognitive developmental delays, academic difficulties, and psychopathology. This study tested whether markers of emotion dysregulation in infancy predict emotion dysregulation in toddlerhood, and whether those associations depended on maternal sensitivity. When children (*N* = 111) were 7 months, baseline respiratory sinus arrhythmia (RSA), RSA withdrawal, and distress were collected during the Still Face Paradigm (SFP). Mothers’ reports of infant regulation and orientation and maternal sensitivity were also collected at that time. Mothers’ reports of toddlers’ dysregulation were collected at 18 months. A set of hierarchical regressions indicated that low baseline RSA and less change in RSA from baseline to stressor predicted greater dysregulation at 18 months, but only for infants who experienced low maternal sensitivity. Baseline RSA and RSA withdrawal were not significantly associated with later dysregulation for infants with highly sensitive mothers. Infants who exhibited low distress during the SFP and who had lower regulatory and orienting abilities at 7 months had higher dysregulation at 18 months regardless of maternal sensitivity. Altogether, these results suggest that risk for dysregulation in toddlerhood has biobehavioral origins in infancy but may be buffered by sensitive caregiving.

Development of emotion regulation is a crucial accomplishment of early childhood. Early emotional experiences and attempts to regulate those experiences serve important developmental functions for an infant (Abe & Izard, 1999). Some of these functions are accomplished through synchronized dyadic interactions between caregivers and infants, formation of attachment bonds, and early social referencing behaviors (Abe & Izard, 1999). Failure to develop emotion regulation abilities is associated with social and cognitive developmental challenges (Eisenberg et al., 1998; Morris et al., 2007; Penela et al., 2012), academic difficulties (Graziano et al., 2007), maladaptive behavior such as aggression or withdrawal later in childhood (Calkins et al., 1998; Hill et al., 2006), and psychopathology (Aldao et al., 2010; Berking & Wupperman, 2012; Keenan, 2000; McLaughlin et al., 2011). Current research tends to focus on single markers of emotion dysregulation at one time point. However, consistent with a developmental psychopathology perspective, the development of emotion regulation and dysregulation invariably occurs within a multilevel system (Adrian & Berk, 2020; Adrian et al., 2011). The developmental process of acquiring emotion regulation skills is dependent on both innate infant characteristics, such as physiological arousal, behavioral affect, and an ability to orient and self-soothe (Calkins et al., 2002; Santucci et al., 2008), as well as external markers, such as maternal sensitivity (Crockenberg & Leerkes, 2004; Fox & Calkins, 2003). Therefore, there is a need to better understand how both infant and caregiver influences affect child emotion dysregulation. Learning more about these processes, beginning in infancy, may increase our ability to detect risk for psychopathology much earlier in development (Mittal & Wakschlag, 2017).

## Defining emotion dysregulation in early childhood

Emotion dysregulation is not merely the absence or opposite of emotion regulation (Beauchaine et al., 2020; Cole et al., 2004, 2017). Emotion regulation involves activation of a goal to influence emotions (conscious or automatic). Although some forms emotion regulation can emerge early in development (e.g., thumb sucking to self-soothe; Crockenberg & Leerkes, 2004; Eisenberg et al., 2010), most forms of emotion regulation are developed through socialization and coregulation, which become more sophisticated and require more cognitive ability as development progresses (Vohs & Baumeister, 2016). In contrast, early signs of emotion dysregulation may appear as simple expressions of emotion, such as distress, that is more intense than typical or age normative. Therefore, emotion dysregulation may arise and be detected earlier in development than emotion regulation (Beauchaine et al., 2020). In this way, markers of emotion dysregulation may be particularly useful to observe during infancy to predict future dysregulation.

Despite the importance of studying emotion dysregulation in infancy, no consensus has been reached regarding its conceptualization (Webb et al., 2020). Definitions have ranged from wide: “the failure to meet the developmental tasks of emotional development” (Cole et al., 1994, p. 77) to more detailed: “an inability to respond to stimuli with well-maintained control” (Keenan, 2000, p. 420). For this paper, we define infant emotion dysregulation as the inability to maintain emotional control, engage with the environment, or recover from distress in a developmentally appropriate manner.

## Infant markers of emotion dysregulation

### Physiological stress response

One marker of infant emotion dysregulation may be physiological stress reactivity and regulation (Davis et al., 2011). Parasympathetic nervous system (PNS) activity has been researched broadly among child, adolescent, and adult samples (Beauchaine, 2001, 2015b; Porges et al., 1994) but has received comparatively less empirical attention in infants compared to other stress response pathways (e.g., infant hypothalamic-pituitary-adrenal axis; Davis et al., 2011). Respiratory sinus arrhythmia (RSA) is one measure of PNS functioning (Porges, 2007) and is an established correlate of mental health symptoms (Beauchaine, 2001; Wagner & Waller, 2020). RSA is a measure of the rhythmic increases and decreases in heart rate that occur across the respiratory cycle due to vagal (i.e., parasympathetic) influences on heart rate (Cacioppo et al., 1994). RSA responses likely represent an infant’s emerging ability to cope and self-regulate in response to a stressor (Hinnant & El-Sheikh, 2009; Porges, 2007). During a threatening or challenging situation, the body withdraws vagal influence to allow for active responding. In the absence of a strong sympathetic nervous system influence, this withdrawal (leading to a decrease in RSA from baseline) often correlates with a higher heart rate. Vagal withdrawal also typically corresponds to shifting attentional and behavioral resources in response to threat or challenge and increased attention to the environment, which allow the individual to confront a threatening situation (Porges, 2007). Vagal engagement (leading to increases in RSA from baseline) typically corresponds to slower heart rate and can help facilitate a return to homeostasis (Brooker & Buss, 2010; Porges, 2007). Higher baseline RSA may reflect more positive and responsive emotional states in infancy while lower baseline RSA has been related to more adjustment and cognitive problems (Stifter & Corey, 2001). Decreases in RSA in response to stress (i.e., RSA withdrawal) may reflect more positive and responsive emotional states in infancy and better regulatory abilities (Calkins & Hill, 2007; Moore & Calkins, 2004). In contrast, smaller decreases in RSA from baseline to a stressor or increases in response to stress (i.e. RSA augmentation) have been related to more adjustment and cognitive problems in some studies (Graziano & Derefinko, 2013). It is thought that healthy RSA responses include returning to prestress, baseline levels after the stressor is removed (i.e., during recovery; see Porges & Furman, 2011, for a review).

### Behavioral affect

A second possible infant marker of infant emotion dysregulation is infant behavioral affect. Negative emotionality is a component of temperament that can reflect patterns of intense, distressed emotions (Buss & Plomin, 1986; Kim & Kochanska, 2012). It can be used as an index of how quickly, strongly, and regularly the infant experiences negative emotions (Rothbart & Derryberry, 2002). Negative affect is hypothesized to interfere with healthy emotion regulation in two ways. First, prolonged negative emotionality overextends neural systems that underlie regulatory capacity, weakening their ultimate efficiency (Patterson et al., 2016). Secondly, children who become overwhelmed by negative emotions likely have experienced failures to regulate their highly negative affect in the past, thus decreasing the chance that they will attempt to regulate in the future (Calkins & Degnan, 2006; Petrenko et al., 2019). Empirical studies support this hypothesis. O’Connor (2001) found that negative affect in infants at 12 months was associated with the development of depressive symptoms at 6 years of age (O’Connor, 2001). Similarly, Bridgett et al. (2009) discovered that high levels of negative affect in early infancy were associated with less ability to orient and regulate at 4–12 months (Bridgett et al., 2009). Finally, multiple researchers have shown that higher negative affect in infancy predicts lower effortful control in toddlers and preschoolers (for a review, see Lonigan et al., 2004).

### Orientation and regulation

A third potential marker of emotion dysregulation in infancy concerns infant orientation and regulation, or the ability to focus attention and regulate behavior (Stifter & Dollar, 2016). The ability to focus may include behaviors such as sustained attention and pleasure at observing an object. Regulating may include behaviors such as enjoyment while being held by the caregiver or a reduction in crying or distress when the caregiver uses soothing techniques such a patting or rubbing their back. Because orientation and regulation may be one of the earliest manifestations of attention and regulatory functioning (Gartstein et al., 2013; Petrenko et al., 2019), deficits in this area could be an early marker of emotion dysregulation. Orientation and regulation in infancy provide a foundation for more effortful self-regulation later in childhood (Kochanska et al., 2000; Putnam & Rothbart, 2006). In turn, effortful control, or the child’s capacity to use attentional resources and inhibit behavioral responses in a way that can regulate their emotion (Rothbart et al., 1994), has been linked to later forms of emotion regulation (Petrenko et al., 2019). Low infant orientation and regulation have been associated with several maladaptive outcomes across the lifespan (Bridgett et al., 2015; Petrenko et al., 2019).

## Caregiver associations with infant emotion regulation

During the first year of life, infants rely heavily on caregivers to aid with coregulation of their distress (Spangler et al., 1994). Infants can use behavioral cues such as facial expression, crying, tone of voice, and gestures to communicate (Weinberg & Tronick, 1994). Sensitive caregivers can recognize and respond to cues accurately, warmly, and in a timely manner (Ainsworth et al., 1978, 2015) facilitating the infant’s ability to manage and express their emotions effectively (Landry et al., 2006). Over time, infants and young children can learn about their emotions and how to respond to them by observing and modeling the expressiveness of a sensitive caregiver (McCoy & Raver, 2011). This process is central to the development of emotional regulation skills during early childhood. Infants and young children are highly attuned to the emotional cues of their caregivers from early on (Grossmann et al., 2005). Through repeated interactions, they begin to recognize patterns in facial expressions, tone of voice, and body language that convey various emotions (Thompson, 2014). A sensitive caregiver, who responds promptly and appropriately to the child’s emotional signals, provides a rich emotional environment for the child to learn from (Davidov & Grusec, 2006). By observing how their caregiver expresses and regulates emotions in different situations, children start to internalize these strategies and develop their own repertoire of emotional regulation skills (Eisenberg et al., 1998). For example, if a caregiver calmly soothes a distressed child, the child may learn to self-soothe by imitating similar behaviors or seeking comfort from others when feeling upset (Denham et al., 1994). Moreover, the modeling of emotional expressiveness by a sensitive caregiver helps children to understand the range and complexity of emotions (Halberstadt et al., 2001). They learn that it is normal to experience a variety of feelings and that emotions can be expressed in adaptive ways (Morris et al., 2007). This understanding lays the foundation for developing empathy towards others and navigating social interactions effectively (Denham, 1998). Through this attachment-mediated and social learning process of observation, imitation, and internalization, all within the context of sensitive parent-infant interactions, children gradually acquire the ability to recognize, label, and regulate their emotions more autonomously (Eisenberg et al., 2010). The presence of a securely attached, sensitive caregiver who provides a nurturing and responsive environment significantly contributes to this emotional development journey, fostering the emergence of adaptive emotional regulation skills that serve the child well into adulthood (Belsky & Fearon, 2002). Sensitive caregiving in infancy has been associated with better physiological regulation (Perry & Calkins, 2018), reduction of infant distress (Kopp, 1989), fewer behavioral problems, and higher levels of emotion regulation overall (Gunnar & Donzella, 2002; Leerkes et al., 2009). Because of this dependence on caregivers for the regulation of infant emotional states during the first year, caregiver behaviors during this time can greatly shape the development of infant emotion regulation.

High maternal sensitivity may help reduce maladaptive emotional development. Crockenberg and Leerkes (2003) found that highly sensitive mothers may buffer the risk of later anxious behavior for highly reactive infants. Sensitivity as a protective factor have been explored with infants in multiple studies (Haley & Stansbury, 2003; Martinez-Torteya et al., 2014; Porter et al., 2003; Schore, 2001). Caregiver sensitivity has also been identified as a buffer for negative outcomes in toddlers and young children (Bernier et al., 2010; Eisenberg et al., 2005; Gaertner et al., 2008; Leerkes et al., 2009; Spinrad et al., 2004, 2007). For example, Penela et al. (2012) found that temperamental fear and poor socioemotional development was reduced with high-quality caregiving. Additionally, children whose mothers were high in sensitivity in the first 2 years of life were less likely to demonstrate behavior problems - a correlate of adaptive emotion regulation - at ages 2 and 3 years (NICHD Early Child Care Research Network, 1998).

Conversely, children who experience less sensitive caregiving may be at greater risk of maladaptive development. Parents who are less responsive to infants’ distress may have infants who experience prolonged distress, which may compromise any attempt the infant makes at regulating their own emotions (e.g., Bridgett et al., 2009; Patterson et al., 2016; Stifter & Spinrad, 2002). This prolonged distress may weaken the connectivity of neural systems that support response inhibition, such as the ventrolateral prefrontal cortex, dorsolateral prefrontal cortex, medial frontal cortex, and parietal cortex (Patterson et al., 2016). Additionally, infants who do not expect responsive caregiving based on past experiences may experience excessive activation of other stress response systems, such as the sympathetic nervous system, HPA axis, and cardiovascular system, which can contribute to greater wear and tear on the body and worse health (Enlow et al., 2014; Obrist, 1976). These studies suggest that when examining the development of infant emotion regulation, it is crucial to examine caregiver influences (Petrenko et al., 2019).

## Interactions between infant markers and caregiver sensitivity

Interactions between infant characteristics and maternal sensitivity may be associated with differences in emotion regulation (Crowell et al., 2009; Thomas et al., 2017). High caregiver sensitivity can buffer the risks of high emotional reactivity. For example, among infants with greater negative temperament, those who experienced higher maternal sensitivity had a decreased chance that they would be emotionally dysregulated in later childhood (Crockenberg & Leerkes, 2006). Additionally, infants who had difficulty regulating fear displayed internalizing behavior as toddlers, but only if they experienced low caregiver sensitivity. High caregiver sensitivity functioned to buffer that link (Early et al., 2002; Penela et al., 2012). Multiple studies have demonstrated that, among children with high emotionality, those whose caregivers were less sensitive exhibited poorer regulation, and those whose caregivers had high sensitivity displayed the best regulation skills (Kim & Kochanska, 2012; Pluess & Belsky, 2011; Roisman et al., 2012).

In contrast, low caregiver sensitivity may serve as a particular risk factor for infants who lack rudimentary emotion regulation skills. For example, among infants who demonstrated higher levels of negative affect, low caregiver sensitivity was related to less attention regulation and more avoidant behaviors (Thomas et al., 2017). Lower levels of caregiving may also increase the risk of maladjustment, particularly when the infant’s regulatory skills are low (Wu & Feng, 2020).

## Developmental psychopathology perspective

Several researchers in the field agree that emotion dysregulation, being a complex, multifaceted, and dynamic construct, should be studied using a multimethod approach in order to capture a complete understanding of its development and the mechanisms involved (Adrian & Berk, 2020; Beauchaine et al., 2020; Crowell et al., 2020; Rogers et al., 2014; Strauman, 2017; Wakschlag et al., 2010; Webb et al., 2020). Rather than resulting from one discrete emotional component, emotion regulation is the reciprocal interaction between multiple influences that creates emotional activation and regulation (Thompson, 2011a). These different systems, rather than always converging, may be continually and mutually interacting, becoming more integrated as the individual matures. Therefore, rather than studying infant emotion dysregulation using one method in isolation, researchers using multiple methods within the same sample would be in a better position to capture the complexity of the construct (Adrian & Berk, 2020).

However, very few studies to date have used more than one method to assess emotion dysregulation in young children (Webb et al., 2020). Adrian et al. (2011) conducted a 35-year review of emotion regulation assessment, including those used with infants. Out of the 157 studies in 42 major journals measuring emotion regulation, 61.1% used only one method of measurement, 23.6% used two methods, 10.8% used three methods, and 4.5% used four methods (Adrian et al., 2011). Webb et al. (2020) argued that each measurement category has its own unique strengths and limitations. Given this, as emotion regulation is defined by the methods used to assess it, they recommended using a multimethod approach, which will provide a more comprehensive view of the construct. Furthermore, the majority of these and other emotion regulation and dysregulation studies focused on toddlers and older children rather than infants (Adrian et al., 2011; Petrenko et al., 2019). Petrenko et al. (2019) observed that most research focuses on the development of regulatory capacity in toddlers and older children. However, many regulatory behaviors in infancy precede those in toddlerhood (such as orienting and regulation preceding effortful control). Thus, they contended that it is crucial to study these key regulatory behaviors in infancy, as those behaviors are foundational to the development of later regulation.

## Current study

Embracing a developmental psychopathology perspective, the goal of this study was to explore the development of infant emotion dysregulation by using a multidimensional approach. Three major markers of emotion dysregulation in infancy were explored to discover which markers contributed to the development of emotion dysregulation in toddlerhood. Although dysregulation changes across development, research has shown stability between early-life regulatory problems (e.g., excessive crying, sleep and feeding difficulties) and later childhood dysregulation (e.g., negative emotionality, conduct problems, hyperactivity; Crowell et al., 2015; Winsper & Wolke, 2014). The presence of multiple regulatory problems, including excessive crying, sleep and feeding difficulties, is conceptualized as “dysregulation” in toddlerhood and is linked to negative outcomes (Hemmi et al., 2011; Winsper & Wolke, 2014). The current study also investigated which of these markers may be moderated by maternal sensitivity, indicating possible intervention opportunities.

Our first aim was to examine whether biobehavioral markers of emotion dysregulation during infancy predict emotion dysregulation a year later when children are toddler. We hypothesized that lower baseline RSA during infancy would predict higher levels of toddler emotion dysregulation (Hypothesis 1a), less RSA withdrawal during infancy would predict greater toddler emotion dysregulation (Hypothesis 1b), higher levels of distress during infancy would predict greater toddler emotion dysregulation (Hypothesis 1c), and lower levels of orienting and regulation during infancy would predict greater toddler emotion dysregulation (Hypothesis 1d).

Our second aim was to test whether the associations between these four markers of emotion dysregulation during infancy and toddlers’ emotion dysregulation are moderated by maternal sensitivity. We hypothesized that maternal sensitivity would buffer infants who exhibited risk for emotion dysregulation at age 7 months (Hypothesis 2). In other words, the associations articulated in Hypotheses 1a-1d would only be significant for infants who experienced low sensitivity. In contrast, markers of emotion dysregulation during infancy were not expected to be associated with toddler emotion dysregulation when mothers were highly sensitive.

## Methods

### Participants

Data for this study were drawn from a prospective, longitudinal study exploring the intergenerational transmission of emotion dysregulation during the prenatal and early postnatal periods. For the present study, data were drawn from 111 mother-infant dyads during 7- and 18-month assessments. Infants were 47% female (53% male). The majority (65.5%) of infants were White, 34.9% Hispanic/Latine, with 10% or less of other racial/ethnic groups. Fifty-nine percent of women had a household income of $50,000 or more per year, 17% were between $30,000 and $49,000, and 22% made $29,000 or less per year in their household. Fifty-six percent of women were college graduates or had post graduate education, 42% were high school graduates and may have had some college experience, and 2 percent had not graduated high school. Most women were married (79%), 15% were single, never married, and 6% were separated or divorced.

### Procedures

All women provided a written informed consent before every assessment and digitally before completing online questionnaires. Women were oversampled for emotion dysregulation. (More details about recruitment, study design, eligibility criteria, and assessment procedures can be found in Lin et al., 2019.) All study procedures were approved by the University of Utah and Intermountain Medical Center Institutional Review Board.

We obtained RSA data from dyads during the still-face paradigm at 7 months. Infant regulation/orientation data from mother report were obtained from online questionnaires. We were also able to code behavioral distress from these infants, and maternal sensitivity data from these mothers. Missing data were due to video being unscorable (e.g., poor camera angle) or video or software malfunction. Of these numbers, 86% completed follow-up questionnaires when their infants neared 18 months of age. Attrition occurred due to families moving, mothers declining to continue, or being unable to reach the family after repeated attempts.

The 7-month and 18-month timepoints were chosen because at these ages infants and toddlers are learning important regulatory skills, in part via interactions with their primary caregivers. Furthermore, these timepoints align with existing research, which allows us to more easily compare findings across studies and build upon previous knowledge (Braungart-Rieker et al., 2014; Kiel & Kalomiris, 2015; Peltola et al., 2015; Aktar & Pérez-Edgar, 2020; Bridgett et al., 2011; Mangelsdorf et al., 1995). By aligning with existing research, we felt it would be easier to compare findings across studies and build upon previous knowledge.

The most prominent was the cognitive and emotional development of the young children. By 18 months, the child has undergone significant developmental changes, including language acquisition, social cognition, and self-regulation. We wanted to capture these developmental changes and their influence on other variables of interest to us in the study.

Prior to the 7-month visit, women completed a series of questionnaires online (mean infant age = 6.61 months, *SD* = .93). The survey included questions regarding the participant’s age, socioeconomic status, educational background, and both mother’s and baby’s race and ethnicity.

Mothers also completed the Infant Behavior Questionnaire (IBQ; Gartstein & Rothbart, 2003; Putnam et al., 2014), which is a questionnaire measure of infant temperament, at this time. Mothers received a link to the measures via Research Electronic Data Capture (REDCap) email before attending the 7-month lab visit. Researchers attached heart rate and respiration monitoring equipment on both mothers and infants during lab visits.

### Still-face paradigm and free play

Following a baseline assessment, infants were placed in a highchair and the experimenters introduced the Still-Face Paradigm (SFP; Haley & Stansbury, 2003; Tronick et al., 1978), which is a validated experimental procedure developed to assess socioemotional regulation in infants (Giusti et al., 2018). During the SFP, mothers interact with their infants in a play, still-face, and recovery episode (Mesman et al., 2009; Moore & Calkins, 2004; Tronick et al., 1978).

The procedure was stopped if the infant became too upset, such as uninterrupted crying for 10–15 s, or if the mother requested a stop on the procedure. Continuous measures of heart rate and RSA were collected during the still-face paradigm. At the end of the 7-month laboratory visit, mother-infant dyads engaged in a free play interaction with their infants. Mothers were instructed to play with their infants as they normally would on a carpet for five minutes without toys and 10 minutes with age-appropriate toys that were provided.

### Follow-up assessment

When infants neared 18 months old, their mothers (regardless of whether they attended the 7-month visit) were contacted via email or phone and invited to complete online questionnaires (via RedCap software) prior to a lab visit. From the 18-month visit, only data from the online questionnaires were used for this analysis. Among these online questionnaires was the Infant-Toddler Social and Emotional Assessment (ITSEA).

### Measures

#### Infant respiratory sinus arrhythmia (RSA)

Electrocardiogram data were collected from infants using a two-lead configuration with spot electrodes placed on the right clavicle and left ribcage (Qu et al., 1986) using MindWare mobile devices (MindWare Technologies Ltd., Gahanna, OH; Biolab software version 3.1). Respiratory sinus arrhythmia (RSA) was defined as the high frequency band of the power spectrum waveform (0.24–1.04 Hz for infants) and was scored in 30-second epochs by trained research assistants using Mindware’s heart rate variability analysis software. This software flags peaks of the R wave within each QRS complex (the graphical depiction of a heartbeat) and records the interval between adjacent R peaks (i.e., interbeat intervals). The software identifies whether the interbeat intervals are within an expected range for the time series, as well as whether they are within expected deviations considering surrounding data. Trained research assistants reviewed the flagged R peaks and made corrections when necessary (i.e., misidentified R peak). Epochs were considered missing if: (1) there were ≥ 5 s of missing or unusable data or (2) RSA values fell outside of the expected range of 1–10. RSA data were double-checked by a senior investigator when necessary. Infant RSA was collected during the SFP. Baseline scores were calculated by averaging the RSA values during the first episode of the SFP (i.e., baseline play episode). Change scores were calculated by subtracting the mean RSA of the first episode (baseline play) from that of the second episode (still-face). Therefore, lower scores reflect greater withdrawal (more decrease in RSA from baseline to task, i.e., lower scores reflect lower values).

#### Infant behavioral distress

Infants’ affect and attention during the still-face episode of the SFP were coded in 1-second intervals by trained research assistants using the Infant and Caregiver Engagement Phases coding (Weinberg & Tronick, 1999). Infants’ behavior was assigned one of seven mutually exclusive codes using Noldus Observer: protest, withdrawal, negative engagement, engagement with an object or the environment, social monitor, positive social engagement, or unscorable. For the purposes of predicting emotion dysregulation, three codes: negative engagement, protest, and withdrawal were added to create a measure of “infant distress,” which was used in this analysis. Coders were blind to all other mother-infant data.

Proportion scores for each of the codes were calculated and ranged from 1.00 (indicating the infant engaged in that behavior 100% of the time) and 0 (indicating the infant did not engage in that behavior). Thirty-one of the observations were double-coded, and the interrater reliability (intraclass correlation) for the measure of infant distress during the still-face episode was .996.

#### Infant behavior questionnaire (IBQ): regulatory control/orienting

Mothers completed the Infant Behavior Questionnaire Revised Short Form (IBQ-R; Gartstein & Rothbart, 2003; Putnam et al., 2014) online with other questionnaires at the 7-month time point. The IBQ-R Short Form consists of 91 items and 14 scales and is a parent-report measure of infant temperament, based on the IBQ developed by Rothbart (1981). Mothers rated the frequency that their infant engaged in specific day-to-day behaviors in the prior one to two weeks using a 7-point scale, with responses ranging from 1 (*never*) to 7 (*always*). The Orienting/Regulation dimension, which is used in this analysis, includes the subscale scores of low intensity pleasure, cuddliness, duration of orienting, and soothability. Each of these subscales includes 6–7 items, such as “When rocking your baby, how often did s/he take more than 10 minutes to soothe?” This dimension had a good internal consistency (Cronbach’s alpha = .75; Putnam et al., 2014).

#### Maternal sensitivity at 7 months

Video-recorded interactions during the two nondistressing play episodes were coded using five-point scales capturing sensitivity, intrusiveness, detachment, and positive regard. These scales were adapted from the Observational Record of the Caregiving Environment and (NICHD Early Child Care Research Network, 1998.) The sensitivity scale assessed the mother’s ability to follow her infant’s lead by responding appropriately to the infant’s signals. The intrusiveness scale assessed the mother’s engagement in psychologically or physically directive behaviors. The detachment scale assessed mothers’ lack of emotional engagement and awareness of infants’ needs. The positive regard scale assessed mothers’ expressions of positive affect and delight directed toward the infant during the interaction. Researchers assigned ratings based on both quality and quantity of mother behavior using a 1–5 scale with: 1 = Not at all characteristic, to 5 = Highly characteristic. All videos were double coded by trained coders, and disagreements greater than one scale point were conferenced. For cases with disagreements that were less than one scale point, the average scores were used in the analyses. Intraclass correlations for the ratings of caregiver behavior were between .56 and .83. A composite measure of overall maternal sensitivity during the play interactions was created by averaging the four ratings (after reverse scoring maternal intrusiveness and detachment; *α* = 0.61).

#### Emotion dysregulation in toddlers

Emotion dysregulation in this study was measured at 18 months using the Infant-Toddler Social and Emotional Assessment (ITSEA; Carter et al., 2003). The ITSEA is a caregiver-report tool that measures social-emotional problems and competencies in children 12–35 months old (Carter et al., 2003). Mothers completed this form when their infants neared 18 months of age. For this analysis we used the Dysregulation domain, which consists of 4 subscales (Negative Emotionality, Sleep, Eating, and Sensory Sensitivity) and 34 items. Some example items include: “Cries or has tantrums until he or she is exhausted,” “Is impatient or easily frustrated,” and “Is whiny or fussy when he or she is NOT tired.” The dysregulation domain had an acceptable Cronbach’s alpha of .66.

### Analytic plan

The percentage of missing values ranged from 0 for some variables to as high as 21.62% for 7-month observed distress, and only about 78% of the participants in the sample would have been available for analysis under the listwise deletion method. Data are missing due to non-response, attrition, equipment failure, infant distress, or family move. No discernible pattern appeared after checking for missing data patterns via SPSS. Those who atritted did not differ in demographics or key variables assessed at 7 months. We addressed the problem of missing data using the multiple imputation technique including all analysis variables under the assumption that variables are missing at random (Schafer & Graham, 2002). SPSS’s multiple imputation command generated 80 imputed datasets and 500 iterations (Van Buuren, 2018). Datasets were pooled and analysis run on imputed dyadic data (*N* = 111). Imputed values compare reasonably to observed values, and results using listwise deletion are similar to multiple imputation, so imputed regression results are presented (Manly & Wells, 2015). For ease of investigating simple slopes, the bar procedure was used as pooling technique. This procedure was created by Baranzini (2018) and facilitates the compression of several imputed data frame outputs into a single imputed data frame. The bar procedure pools and creates a mean of the multiply-imputed values as they are generated by the algorithm, retaining between-sample variability in the estimates. This procedure has also been used by other researchers (Orkorn, 2022; van der Kamp et al., 2020).

Five hierarchical linear regressions were conducted. Each of the first four regressions focused on one of the four measures of early emotion dysregulation. A fifth regression was conducted to determine whether significant effects held independently of one another. This fifth regression included all significant main effects and interactions, which included observed infant distress, mother-reported orienting and regulatory control, baseline RSA-maternal sensitivity interaction, and RSA withdrawal-maternal sensitivity interaction. Demographic variables were screened for possible inclusion in Step 1 of the hierarchical regression as covariates but were ultimately not included because of non-correlation with study variables. Step 2, testing for main effects, included the individual infant predictor (baseline RSA, RSA withdrawal, infant distress, and infant orienting and regulation), mean-centered. Step 3 included maternal sensitivity, mean-centered. Step 4, testing for an interaction between the predictor and maternal sensitivity, included a variable created by multiplying the predictor and the maternal sensitivity variable. Significant interactions were probed at three levels: −1 standard deviation (*SD*), mean, and + 1*SD*, using an online computational tool (Preacher et al., 2006), to determine simple slopes.

## Results

Prior to conducting primary analyses, demographic characteristics of participants were assessed. Correlations and descriptive statistics for predictor and outcome variables Table 1 and Table 2.

### Model 1: Infant baseline RSA predicting dysregulation

**Figure 1.**
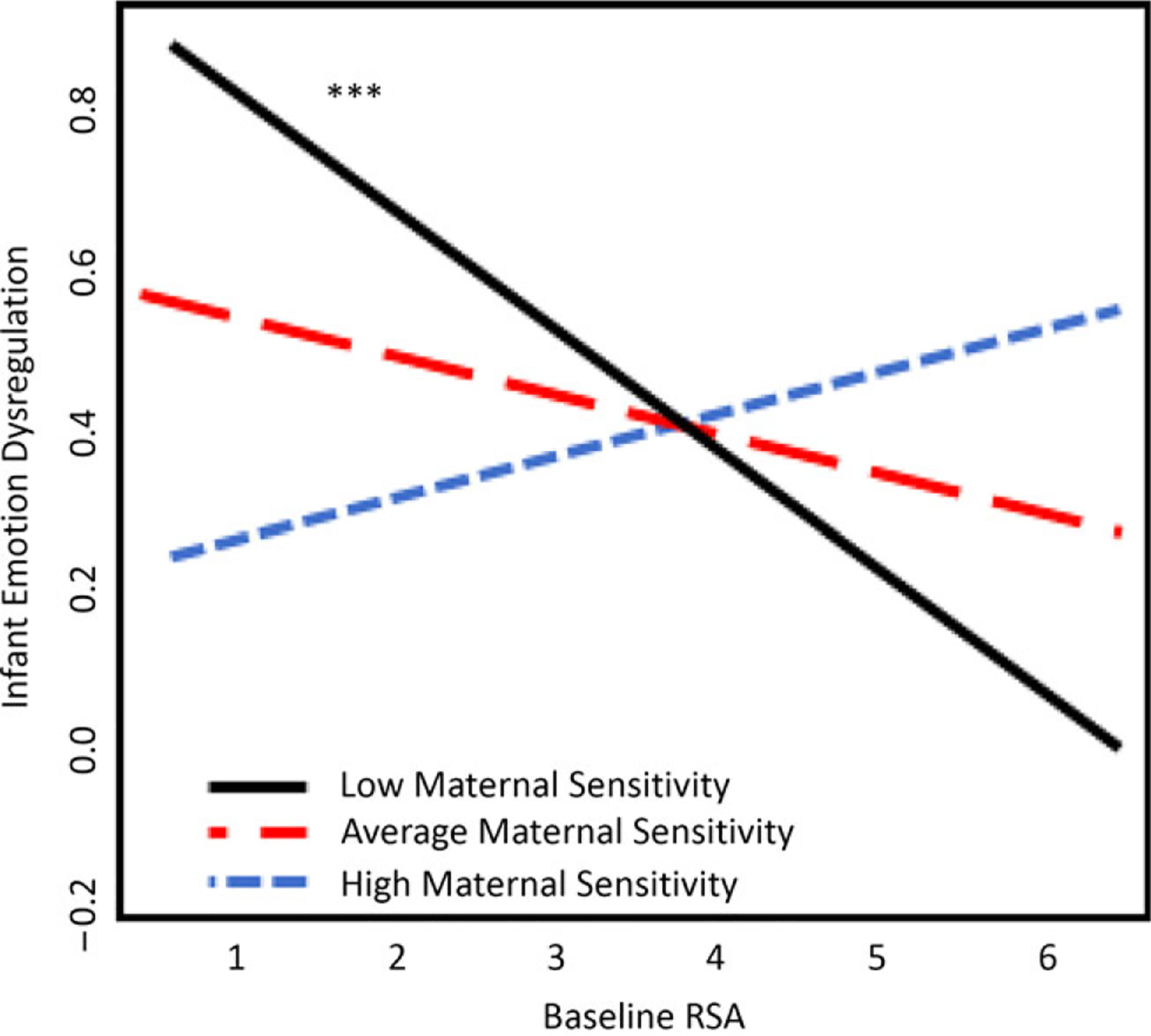
Baseline RSA and maternal sensitivity interaction plot. Independent variables were centered for graphical representation. Results from Model 1 show significance only for infants who have experienced low maternal sensitivity; ***p < .001 (two-tailed).

Results of the first linear regression model indicated that neither baseline RSA nor maternal sensitivity were significantly associated with toddler emotion dysregulation as measured by the ITSEA (see Table 3). However, there was a significant interaction between baseline RSA and maternal sensitivity at 7 months (*β* = .26, *t* = 2.69, *p* = .008; See Table 3). Simple slopes analyses showed low baseline RSA at 7 months predicted higher dysregulation at 18 months, but only for infants whose mothers had low sensitivity (*B* = −.09, *t* = −2.26, *p* = .03; Fig. 1). The associations between baseline RSA and toddler emotion dysregulation were not significant for infants with average and high maternal sensitivity (average: *B* = −0.05, *t* = −1.61, *p* = .11; high: *B* = .05, *t* = 1.30, *p* = .20).

### Model 2: Infant RSA withdrawal predicting dysregulation

**Figure 2.**
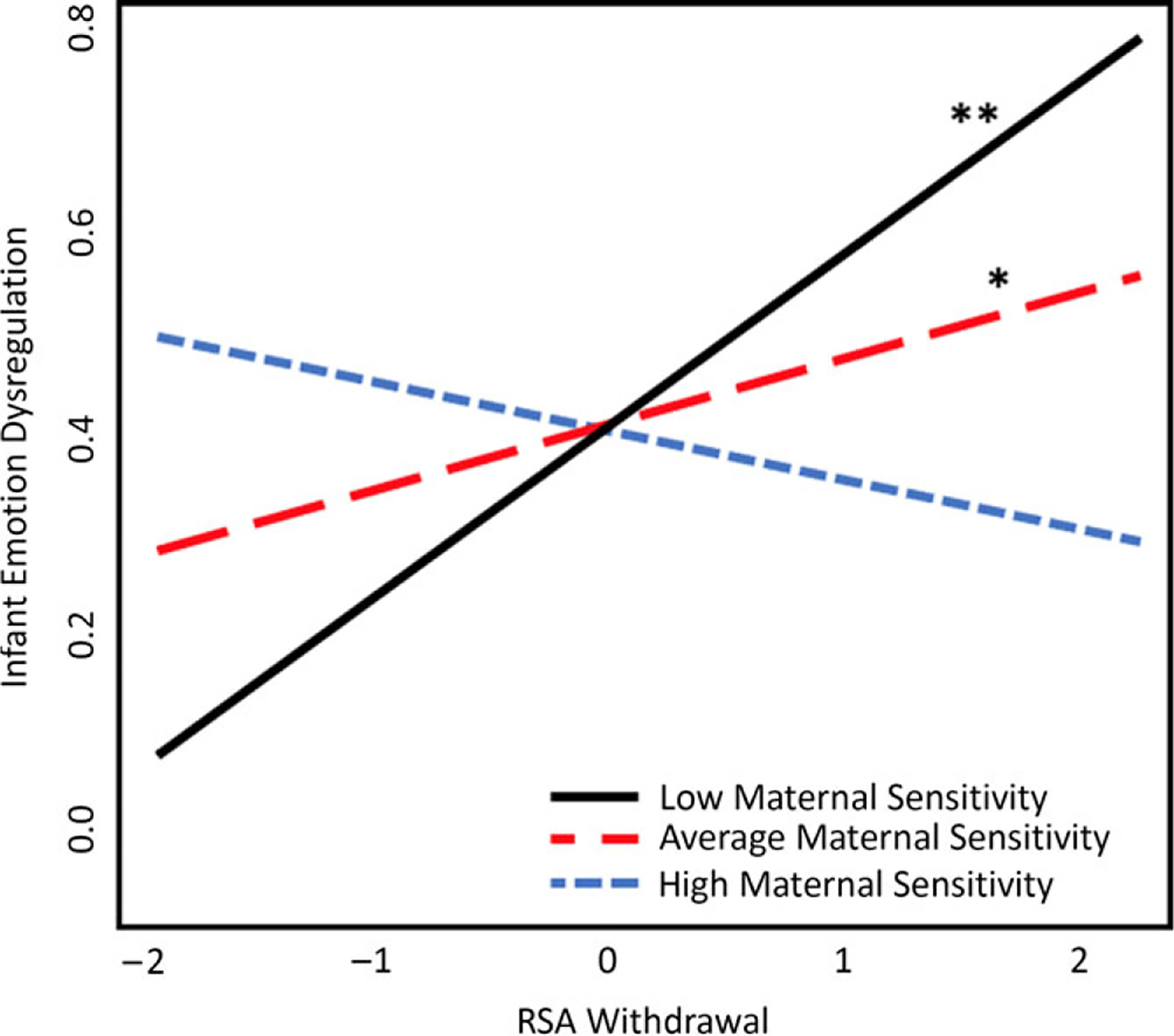
RSA withdrawal and maternal sensitivity interaction plot. Independent variables were centered for graphical representation. RSA withdrawal = still-face task mean minus baseline play mean. Lower scores represent greater withdrawal, or more decrease from baseline to task. Higher scores represent less withdrawal from baseline to task. Because the mean value of RSA change was negative (see Table 3), positive values on the mean-centered *x*-axis reflect actual RSA withdrawal values that are close to zero (reflecting a lack of change in RSA levels from play to still-face episodes) and not positive RSA changes (which would indicate that RSA levels increased from baseline to still-face). Results from Model 2 show significance only for infants who have experienced low maternal sensitivity; *p < .05, **p < .01 (two-tailed). RSA withdrawal = still-face task mean minus baseline play mean. Lower scores represent greater withdrawal, or more decrease from baseline to task. Higher scores represent less withdrawal or increase from baseline to task.

Results of the second linear regression model indicated that neither RSA withdrawal nor maternal sensitivity at 7 months were significantly associated with toddler emotion dysregulation as measured by the ITSEA. However, there was a significant interaction between RSA withdrawal to the still face and maternal sensitivity (*β* = −.26, *t* = −2.88, *p* = .005; see Table 3). Simple slopes analyses showed that lower RSA withdrawal (less change in RSA from baseline to the still-face episode) predicted higher dysregulation at 18 months, but only among infants of mothers with low (*B* = .07, *t* = 3.67, *p* = .0004) or average sensitivity (*B* = .01, *t* = −0.29, *p* = .02; see Fig. 2.) The association between RSA withdrawal and toddler emotion dysregulation was not significant for infants of mothers with high sensitivity (*B* = .05, *t* = −0.92, *p* = .36).

### Model 3: Infant behavioral distress predicting dysregulation

Results of the third linear regression model indicated that higher observed distress during the still-face paradigm at 7 months predicted lower toddler dysregulation at 18 months, contrary to hypothesis 1c (*β* = −.20, *t* = −2.16, *p* = .03). This association was not significantly moderated by maternal sensitivity (see Table 3).

### Model 4: Infant orientation and regulation predicting dysregulation

Results of the third linear regression model indicated that higher levels of mother-reported infant regulation and orientation at 7 months predicted lower levels of toddler dysregulation at 18 months, supporting hypothesis 1d (*β* = −.22, *t* = −2.30, *p* = .02). This association was not significantly moderated by maternal sensitivity (see Table 3).

### Model 5: Including all significant variables in the model

Results of the fifth linear regression model indicated that when the significant main effects of infant dysregulation and interactions with maternal sensitivity were included in the same model predicting toddler dysregulation, only observed infant distress (*B* = −.07, *p* = .04) and RSA withdrawal-maternal sensitivity interaction (*B* = −.08, *p* = .03) remained significant (see Table 3).

## Discussion

Emotion dysregulation in childhood may portend problems with social and cognitive development, academic difficulties, maladaptive behavior such as aggression or withdrawal, and psychopathology later in life (Aldao et al., 2010; Berking & Wupperman, 2012; Calkins et al., 1998; Eisenberg et al., 1998; Graziano et al., 2007; Hill et al., 2006; Keenan, 2000; McLaughlin et al., 2011; Morris et al., 2007; Penela et al., 2012). This study was designed to test whether markers of risk for emotion dysregulation in infancy could be used to predict risk for emotion dysregulation in toddlers. Because of the coregulation that occurs between mother and infant, we tested whether the effect of infant emotion dysregulation on child emotion dysregulation was moderated by maternal sensitivity. Since emotion dysregulation is a multidimensional process, studying it using only one level of analysis may not capture its complexity. Few studies to our knowledge use more than one method of assessing emotion dysregulation, especially in infancy, a time when regulatory processes develop rapidly (Kopp, 1982). Currently, the field is limited by single-variable studies that do not consider the contributions of multiple variables, thereby limiting interpretability and applicability to intervention. Using a multilevel, longitudinal dataset, our study aimed to address these issues. Overall, our results showed that multiple biobehavioral markers in infancy can predict risk for dysregulation in toddlerhood. Our results also suggest that maternal sensitivity may serve as a buffer for infants with certain RSA responses.

### Baseline RSA and maternal sensitivity

We hypothesized that the association between infant baseline RSA and toddler dysregulation would only vary for infants of mothers who displayed lower levels of sensitivity. This hypothesis was supported. Among infants who experienced lower levels of caregiving sensitivity at 7 months, lower baseline RSA predicted higher levels of dysregulation. For infants who experienced average or high caregiving sensitivity at 7 months, baseline RSA was not predictive of dysregulation in toddlerhood. These results suggest that low caregiver sensitivity may be a risk factor for infants with low baseline RSA. Baseline RSA is a marker of infant stress withdrawal and capacity for regulation, and infants with a lower baseline RSA may struggle with physiological aspects of emotion regulation, thus predisposing them to more negative and less responsive emotional states during times of stress (Stifter & Corey, 2001).

Researchers suggest that the level of sensitivity in caregiving may affect children’s regulatory development. Kim and Kochanska (2012) found that infants high in negative affect were more likely to lack regulatory skills when they had less responsive relationships with their mothers (Kim & Kochanska, 2012). Conversely, Spangler (1990) showed that high maternal responsiveness was associated with less “difficult temperament” (e.g., approachability, adaptability, intensity, mood) at 12 months and higher social competence at 2 years (Spangler, 1990). In sum, in the absence of a sensitive caregiver, some infants may struggle to develop effective self-regulatory strategies.

### RSA withdrawal and maternal sensitivity

Like baseline RSA, we hypothesized that the association between RSA withdrawal and later dysregulation would only be significant for infants who experienced lower levels of maternal sensitivity. This hypothesis was also supported by our results. For infants whose mothers displayed average or low sensitivity, lower RSA withdrawal (i.e., a more blunted response) to distress predicted higher dysregulation. As expected, among infants whose mothers were more sensitive, levels of RSA withdrawal were not predictive of later dysregulation.

During the still-face paradigm, infants are expected to show vagal withdrawal leading to lower RSA, and many researchers have established this pattern (Bazhenova et al., 2001; Moore & Calkins, 2004; Weinberg & Tronick, 1996). Higher RSA withdrawal in infants and children has been linked to better soothability, attentional control (Huffman et al., 1998), behavioral distraction (Calkins, 1997), and lower levels of aggression, reactivity, and depression (Perry et al., 2016; Porges et al., 1996). In contrast, less RSA withdrawal has been empirically linked with more dysregulated behavior in infants, particularly in the context of a less sensitive caregiving relationship. Moore and Calkins (2004) showed that less RSA withdrawal was linked to less positive affect and less synchrony in play with their mothers (Moore & Calkins, 2004). As the interaction results in this study suggest, infants who have experienced low or average maternal sensitivity are at particular risk for later dysregulation if they demonstrate less RSA withdrawal. In the context of the still-face, if we assume that less RSA withdrawal in an infant indicates more difficulty regulating emotions, then it follows that those same infants, particularly those whose caregiver was not responsive, would also be more likely to struggle with dysregulation in toddlerhood. Conversely, those infants who display more withdrawal may be demonstrating their ability to effectively respond to a stressor and regulate their emotions in the absence of a sensitive caregiver, making it more likely that they are also able to do this in toddlerhood.

For both baseline RSA and RSA withdrawal, it is possible that higher caregiver sensitivity buffered the effects of physiological dysregulation (i.e., low baseline RSA and low RSA withdrawal). Based on the quality of their past interactions, infants who do not expect or receive sensitive caregiver responses to distress may also experience activation of other stress response systems, such as the sympathetic nervous system (Enlow et al., 2014). This repeated stress response system activation may contribute to “wear and tear” on the body, poor health, and lower ability to effectively cope with stress in the future (Moore et al., 2009). The buffering effect of maternal sensitivity may therefore play an important protective role for infants at risk for maladaptive stress responses. Infants who initially had poorer RSA functioning, but were exposed to high levels of maternal sensitivity, may have demonstrated greater growth in baseline RSA and withdrawal over time, leading to more adaptive emotion regulation.

### Infant behavioral distress

While it is normative and expected for infants to become distressed during the still-face episode, we expected that the infants who become distressed more quickly, more intensely, and for a higher percentage of time, would be more likely to have a higher dysregulation score at 18 months. Instead, we found that higher levels of infant distress in response to maternal unavailability was associated with lower dysregulation at 18 months. For this analysis, only the distress data that were coded during the still-face episode were used. This was the period that the infants’ mothers were not providing their infants with social feedback. We did not analyze distress that was displayed during the reunion period, when mother and infant were given the opportunity to resolve this social stressor. It is possible that, even if the infant was highly distressed, their ability to later regulate and recover during a reunion period may be more predictive of future regulation than the initial reaction to the stressor.

It is also possible that exhibiting distress is a form of regulation that infants use at this developmental stage (Bell & Ainsworth, 1972). Perhaps infants who were able to express their needs (i.e., by communicating distress) were more likely to receive appropriate attention from their caregiver. On the other hand, it is possible that exhibiting low distress in response to the stress of maternal unavailability is a risk factor for later problems with emotion regulation. Infants who displayed less distress during this stressor may have been displaying blunted affect, indicative of withdrawn behavior that is not considered a typical response to the still-face episode in infancy (Guedeney et al., 2013; Tronick & Cohn, 1989). Low distress during the still face is non-normative and may reflect early adversity or poor relational history between mothers and infants.

### Mother-reported infant orienting and regulatory control

As expected, infants whose mothers reported higher levels of orienting and regulatory control at 7 months were less likely to be dysregulated at 18 months. These skills are believed to be one of the earliest types of regulatory functioning that manifests in the infant (Gartstein & Rothbart, 2003; Petrenko et al., 2019; Rothbart & Derryberry, 1981). Regulatory control and orientation are foundational for developing effortful control, which aids the child in using their own attentional resources and inhibit their behaviors in ways that regulate emotions (Rothbart et al., 1994). Infants who are developing these foundational skills by 7 months are more likely to continue an adaptive development in regulatory abilities, thereby being less likely to exhibit symptoms of dysregulation at 18 months. Past research has shown that low regulatory control and orientation early in life have been associated with several maladaptive outcomes across the lifespan (Bridgett et al., 2015; Petrenko et al., 2019).

Contrary to our predictions, the behavioral markers of risk for emotion dysregulation did not significantly interact with caregiving sensitivity to predict emotion dysregulation in toddlers. It may be that these behavioral measures were more robust predictors of emotion dysregulation than the physiological predictors. Physiological markers can be measured at birth or even prenatally, yet behavioral markers, (while they still may appear early in infancy), may be easier to assess in a later stage of regulatory development, reflecting a more embedded part of the infant’s regulatory repertoire. Although physiological traits may be buffered by high maternal sensitivity, it is possible that behavioral traits may persist even when a sensitive caregiver is present.

It is also possible that, since RSA was still maturing at the developmental stage that we were observing, it was particularly amenable to caregiver influence. Polyvagal theory may provide an explanation for this link between autonomic regulation and maternal sensitivity (Porges, 2007; Porges et al., 1994, 1996, 2019; Porges & Furman, 2011). This theory describes neural pathways that regulate autonomic reactivity and show that autonomic state provides a platform for infant-caregiver coregulation. Vagal pathways are bidirectionally linked with areas of the brain that control aspects of social engagement (e.g., facial expression, gaze, vocalizations). This connection has been demonstrated in an intervention study with preterm infants and their mothers. Infants of mothers who engaged in this intervention, which included enhanced maternal care and sensitivity, exhibited enhanced autonomic regulation (Porges et al., 2019). Additionally, improved emotional connection between caregiver and infants may increase the maturity of the vagal regulation system through increased production of oxytocin that comes with increased social interaction (Welch & Ludwig, 2017).

### Strengths, limitations, and future directions

Our study has several strengths, including a longitudinal design and the use of multiple markers of infant emotion dysregulation. However, our findings should be interpreted in the context of limitations. Firstly, 7-month infant orientation and regulation was assessed via caregiver-report. Although the other three measures of infant emotion dysregulation at the 7-month timepoint were either physiological measures or coded by trained lab experimenters, this measure may have been biased or influenced by other factors, including mother’s mood and/or her own level of emotion dysregulation. However, a strength of this mother-report measure is that mothers report on infant behavior across multiple contexts and over time. Secondly, the 18-month measure of infant dysregulation was also a caregiver-report measure. While this measure has been shown to be a reliable measure and predictive of childhood outcomes, it may be more highly correlated with the mother-report of infant orientation and regulation at 7 months because of the effects of common-method variance. Thirdly, we chose to use baseline RSA and RSA withdrawal as our physiological predictors, given that RSA appears to be a reliable and valid peripheral biomarker of emotion dysregulation. However, as some scholars suggest, it is important for future research to include markers from multiple stress response systems, such as preejection period, electrodermal activity, and cortisol regulation patterns, to further examine how complex multisystem activity contributes to the development of emotion dysregulation (Rash et al., 2016; Suurland et al., 2018). Additionally, our sample size is relatively small. Furthermore, although our sample included a sizable proportion of Hispanic/Latine infants, (34.1%), the largest subgroup was White and non-Hispanic/Latine (68.9%). It is unclear whether our findings can be generalized to other samples with different racial and ethnic identities. Lastly, maternal sensitivity was assessed during a play period rather than during a distress episode. This may be a limitation, as some researchers have found maternal sensitivity during distress to be a more salient predictor of affective dysregulation (Leerkes et al., 2009). We noticed that many of our pregnant participants found watching an infant play to be more stressful (as indicated by maternal prenatal sympathetic nervous system responses) than watching an infant cry. We therefore decided to examine maternal sensitivity during the free play episode, rather than sensitivity to infant distress.

## Conclusions

Knowledge of how specific indicators of infant dysregulation in early life confer subsequent risk for later dysregulation is lacking, as is a deep understanding of how caregiving may operate as a moderator in relation to these indicators. This study used a rich, longitudinal dataset that explored the risk of four different indicators of infant dysregulation and the possible moderation of maternal sensitivity. This study elucidates the significance of exploring infancy in the development of emotion dysregulation. We demonstrated that risk for toddler emotion dysregulation can be predicted as early as 7 months. Infants with a certain RSA pattern (low baseline RSA and less RSA withdrawal) may be at risk for toddler dysregulation, but only in the context of low caregiver sensitivity. Higher caregiver sensitivity appeared to buffer this risk. These findings provide guidance on *for whom* early intervention may be most beneficial. Implementing interventions focused on increasing caregiver sensitivity, especially for infants with specific parasympathetic nervous system responses to stress, may reduce risk for the emergence of behavior dysregulation in toddlers. Measurement of multiple markers of emotion regulation and dysregulation in infancy may be important to fully understanding the development of toddler emotion dysregulation.

## Figures and Tables

**Table 1.** Correlations

	1	2	3	4	5	6
1. Observed Distress	−					
2. Orienting/Regulatory control	.16	−				
3. Baseline RSA	.06	− .20*	−			
4. RSA Withdrawal	− .36**	.08	− .18	−		
5. Maternal Sensitivity	− .03	− .17	.05	− .10	−	
6. Toddler Dysregulation	− .20*	− .22*	− .10	.23*	.05	−

Note.

* *p* < .05,

** *p* < .01, (two = tailed.).

**Table 2.** Measures’ descriptive statistics

Variable	*N*	*M*	*SD*
Infant 7-month Measures			
Baseline RSA	111	3.51	.96
RSA Withdrawal	111	−.17	.79
Behavioral distress	111	.85	.39
Regulatory control/orienting	111	5.31	.65
Infant 18-month Measure			
ITSEA: Emotion dysregulation	111	.41	.25
Caregiver Measure			
Sensitivity	111	3.09	.82

*Note. N* = sample size, *M* = mean, *SD* = standard deviation, RSA = respiratory sinus arrhythmia, IBQ = Infant Behavior Questionnaire, ITSEA = Infant-Toddler Social Emotional Assessment.

**Table 3.** Regression results

Model	Hierarchical Regression	Variable	*B*	*SE*	β	*t*	*P*	*R^2^*
1	Step 1	Baseline RSA main effect	− .03	.03	− .10	− 1.07	.29	.01
	Step 2	Maternal sensitivity main effect	.02	.03	.05	.54	.59	.01
	Step 3	Baseline RSA X maternal sensitivity interaction	.08	.03	.26	2.69	.008**	.08
2	Step 1	RSA withdrawal main effect	.07	.03	.23	2.49	.01	.05
	Step 2	Maternal sensitivity main effect	.02	.03	.07	.75	.46	.06
	Step 3	RSA withdrawal X maternal sensitivity interaction	− .10	.04	− .26	− 2.88	.005**	.13
3	Step 1	Infant distress main effect	− .07	.03	− .20	− 2.16	.03*	.04
	Step 2	Maternal sensitivity main effect	.13	.03	.04	.44	.66	.04
	Step 3	Infant distress X maternal sensitivity interaction	.03	.04	.07	.69	.49	.05
4	Step 1	Infant regulation and orientation main effect	− .08	.04	− .22	− 2.30	.02*	.05
	Step 2	Maternal sensitivity main effect	.003	.03	.01	.11	.91	.05
	Step 3	Infant regulation and orientation X maternal sensitivity interaction	− .01	.04	− .03	− .26	.80	.05
4		Infant distress	− .07	.03	− .19	− 2.07	.04*	.11
		Infant orientation and regulation	− .04	.04	− .12	− 1.23	.22	.11
		Baseline RSA X maternal sensitivity interaction	.04	.03	.14	1.50	.14	.11
		RSA withdrawal X maternal sensitivity interaction	− .08	.04	− .21	− 2.23	.03*	.11

*Note. N* = 111; *B* = unstandardized coefficient, *SE* = standard error, β = standardized coefficient, Beta value, *t* = *t* value, *p* = *p* value/2-tailed significance, *R*^2^ = goodness-of-fit measure;

* *p* < .05,.

** *p* < .01,

*** *p* < .001, (two-tailed.).
